# Probiotic Lactobacilli Precautions

**DOI:** 10.3389/fmicb.2019.00375

**Published:** 2019-03-12

**Authors:** José M. Castro-González, Patricia Castro, Hilda Sandoval, Diana Castro-Sandoval

**Affiliations:** ^1^Department of Molecular Biology, Area of Microbiology, University of León, León, Spain; ^2^Hospital de Sierrallana, Torrelavega, Spain; ^3^Natural Sciences Department, Instituto de Enseñanza Secundaria (IES) Eras de Renueva, León, Spain; ^4^European Forum for Primary Care (EFPC), Utrecht, Netherlands

**Keywords:** lactobacilli, probiotics, case report, precautions, safety

Many clinical tests researching into the use of probiotics for the treatment of diseases are now available (Bernardo et al., 2013; Khalesi et al., 2014). However, the vast majority of the research has not been directed toward safety issues, although some potential risks have been described. Some empirical concerns regarding the safety of probiotics are the occurrence of disease, adverse metabolic effects on the gastrointestinal tract and gene transfer events (http://www.fao.org/3/a-a0512e.pdf). This study is focused on some general safety issues such as the antimicrobial resistance of the lactobacilli, the convenience of making a proper identification of these bacteria and on recent risk reports on probiotic lactobacilli in certain risk groups, mainly immunocompromised and patients with short gut syndrome or under cardio-surgery.

## Infection Case Report Involving *Lactobacillus*

The following is a summary of recent case reports of adverse effects seen when using probiotic lactobacilli, the species involved and some comments on each category (Table 1).

**Table 1:** Some adverse effects seen when using probiotic lactobacilli and species involved.

**Adverse effect**	**Case report**	**Species**	**References**
Bacteremia and/or sepsis	Ulcerative colitis AIDS or other immunocompromised patients Pediatric intensive care unit Short gut syndrome and cardio surgery	*L. rhamnosus* *L. acidophilus* *L. casei* *L. rhamnosus*	Vahabnezhad et al., 2013; Meini et al., 2015 Luong et al., 2010; Haghighat and Crum-Cianflon, 2016 Passera et al., 2016 Kochan et al., 2011; Mehta et al., 2013
Pneumonia	RSV bronchiolitis	*L. rhamnosus*	Doern et al., 2014 Conen et al., 2009
Abscesses	Ulcerative colitis Renal transplant End stage renal disease and diabetes Diverse underlying diseases	*L. rhamnosus* *L. casei* *L*. sp. *L. rhamnosus*	Vanichanan et al., 2016 Sherid et al., 2016 Pararajasingam and Uwagwu, 2017
Endocarditis	Bicuspid aortic valve stenosis Mitral insufficiency Aortic and mitral valve replacement Telangiectasia (HHT)	*L. rhamnosus* *L. paracasei* *L. rhamnosus* *L. rhamnosus*	Kato et al., 2016; Norena et al., 2017 Franko et al., 2013 Aaron et al., 2017 Boumis et al., 2018

A few recent cases of bacteremia and/or sepsis associated with lactobacilli have been reported in patients with different underlying diseases such as ulcerative colitis in pediatric (Vahabnezhad et al., 2013) or in adult (Meini et al., 2015) patients suggesting that an extensive damage of the colonic mucous membrane increases the risk of bacteremia. Other cases of bacteremia involve HIV-infected population (Haghighat and Crum-Cianflon, 2016) or other immunocompromised patients (Luong et al., 2010). Three cases of *L. casei* sepsis were described in a pediatric intensive care unit. Two of which with congenital heart disease and the third one with a cervical spinal cord injury (Passera et al., 2016). Kochan et al. (2011) report a case of empyema in a cardiothoracic transplant recipient with a medical history for HIV infection receiving a probiotic containing *L. rhamnosus* GG (LGG). A few casualty reports are described for the first time as one case of sepsis by *L. rhamnosus* in a female aortic heart valve recipient most likely caused by bacterial translocation through a weakened intestinal barrier (Mehta et al., 2013). Another significant first case report was pneumonia after an episode of respiratory syncytial virus (RSV) bronchiolitis secondary to administration of *L. rhamnosus* in an 11-month-old female (Doern et al., 2014).

The incidence of severe infections caused by lactobacilli with abscesses is quite uncommon. The first report of retropharyngeal and spinal epidural abscesses after the consumption of a dairy product containing *L. rhamnosus* was described in a severely immunocompromised patient with active ulcerative colitis (Conen et al., 2009). Another significant clinical report is an intra-abdominal abscess potentially related to probiotics consumption caused by a carbapenem-resistant *L. casei* (Vanichanan et al., 2016). The first link between liver abscess and use of probiotics containing lactobacilli was described recently in an old female patient with a history of diabetes mellitus and end-stage renal disease (Sherid et al., 2016). Other case was a *L. rhamnosus* endocarditis following upper endoscopy in an 80-year-old male frequent consumer of yogurt containing the organism, who required aortic and mitral valve replacement for cure (Pararajasingam and Uwagwu, 2017).

Endocarditis cases due to lactobacilli containing probiotics have also been reported in patients with bicuspid aortic valve stenosis (Kato et al., 2016; Norena et al., 2017) or a mitral insufficiency owing to valvular prolapses (Franko et al., 2013). Other two case reports involved a *L. rhamnosus* endocarditis following upper endoscopy, which required aortic and mitral valve replacement for cure (Aaron et al., 2017). In a systematic review of the literature, authors found ten cases of infective endocarditis caused by *L. rhamnosus, L. casei, L. paracasei*, and *Lactobacillus* spp. apparently linked to a previous use of probiotics. The same authors also described the first case of infective endocarditis caused by *L. rhamnosus* in a patient with hereditary hemorrhagic telangiectasia (HHT) who was a heavy consumer of probiotics (Boumis et al., 2018). HHT is an inherited disorder characterized by malformations of various blood vessels, potentially resulting in bleeding.

The assumption that the arrival of probiotic lactobacilli to the bloodstream producing episodes of sepsis is very unlikely in healthy and asymptomatic individuals and it is an assumed consensus in spite of the high number of bacteria ingested without restrictions.

## Antimicrobial Resistance and Transferability

Currently, information on the antibiotic susceptibility or resistance in probiotics isolated from food is scarce. A review summarizes the current knowledge on antibiotic resistance mechanisms in probiotic bacteria (Gueimonde et al., 2013). Sharma et al. (2014, 2016) published a few reports specifically centered on the prevalent antibiotic resistance of probiotic lactobacilli, which may represent a food safety concern. These authors studied the sensitivity to 45 antibiotics of 19 commercially available probiotic lactobacilli species and/or strains. Most of the isolates exhibited multiple resistance against some commonly used antibiotics. Resistance was especially high toward some relevant antimicrobials, such as nalidixic acid, nitrofurantoin, kanamycin, teicoplanin, cotrimoxazole, amikacin, streptomycin, norfloxacin, vancomycin, and cefepime. It is advisable that new studies on antimicrobial resistance genes be published. In this regard the use of molecular methods and the possibility of comparing whole genomes with reasonably low costs offers new possibilities on this subject (Bennedsen et al., 2011). Recently Campedelli et al. (2018) determined the antibiotic susceptibility patterns of 182 *Lactobacillus* type strains and compared these phenotypes to their genotypes based on genome-wide annotations of antibiotic resistance genes. Most interesting was that many of the species showed antibiotic resistance levels exceeding those recommended by the EFSA. The authors suggested that these cutoff values should be reexamined providing evidence for rationally revising the regulatory guidelines for safety assessment of probiotic strains.

A second important matter is the *in vivo* transfer of resistance determinants to (or from) potentially harmful microbes. The technical difficulties of this kind of studies explains the scarce information available on this subject. It is well known that lactic acid bacteria possess plasmids containing genes conferring resistance to erythromycin, tetracycline, and chloramphenicol, among others (Tannock et al., 1994; Lin et al., 1996; Gevers et al., 2003). The *in vivo* transferability of plasmid-mediated antibiotic resistance between strains of enteric Gram-positive bacteria was studied using gnotobiotic mice associated with the donor species *L. reuteri* (Morelli et al., 2008) and *L. plantarum* (Jacobsen et al., 2007; Feld et al., 2008) carrying plasmids which harbored erythromycin resistance genes and *Enterococcus faecalis* as the recipient strain. The analysis of fecal content showed the *in vivo* transfer of the plasmids. Treatment with erythromycin was a selective pressure that also strongly favored transfer and establishment of a *L. plantarum resistance* plasmid in the gastrointestinal environment (Feld et al., 2008). This could be relevant given the frequent combined use of probiotics and antibiotics.

The transfer by conjugation from enterococci to lactobacilli could occur in the gut of animals. However, the transfer to lactobacilli is quite rare. The first scientific demonstration on an *in vivo* transfer to a *Lactobacillus* probiotic strain was described with regard to a vancomycin resistance gene from an *Enterococcus* strain to *L. acidophilus* (Mater et al., 2008).

Probiotics are frequently used in combination with antibiotics favoring potential transmissible occurrences. However, there is no evidence on lateral gene transfer of antimicrobial resistance *in vivo*, (probably due to technical difficulties) by using no gnotobiotic animals between probiotic lactobacilli and other organisms. The previously mentioned findings support the need for a careful evaluation for probiotics with special consideration in immunocompromised patients or during antibiotherapy. Either way, the scientific information available supports the hypothesis of the existence of a gut resistance gene pool and the possible transferability of antibiotic resistance genes. In either case, reports on possible *in vivo* transfer are very scarce, although they are needed.

## Identification of Probiotic Lactobacilli

A complete safety assessment begins with an appropriate identification of the probiotic strain. Donelli et al. (2013) reported some cases of inaccuracies in phenotype-based identification of strains included within a probiotic product. More recently, Tommasi et al. (2008) for example reported the diagnostic difficulties of *L. casei* bacteremia in immunocompetent patients and a misidentification of *Lactobacillus* spp. as *Leuconostoc* spp. in a clinical case before rectification using a PCR analysis.

Donelli et al. (2013) reviewed common phenotypic and genotypic methods used to differentiate among microorganisms of probiotic interest. These authors conclude that the techniques most commonly used for the typing of probiotic microorganisms are Pulse field gel electrophoresis (PFGE); Random amplified polymorphic DNA-PCR (RAPDPCR); Ribotyping and Amplified fragment length polymorphism (AFLP). The identification of a probiotic strain should be polyphasic and be based on morphological, physiological, and biochemical criteria including some of these previously mentioned and others such as DNA-DNA hybridization, amplified ribosomal DNA restriction analysis (ARDRA), sequencing of the 16S, 23S rDNA and even the whole genome. The genome analysis of LGG variants confirm the relevance for quality assurance and control measures targeting genome stability in probiotic strains (Sybesma et al., 2013).

To sum up, when characterizing clinical isolates and probiotic strains, traditional criteria are not always satisfactory. The comparison needs the use of culture independent molecular-based phenotypic and genomic characterization (Aroutcheva et al., 2016).

## General Comments and Concluding Remarks

The conclusion emitted by the EFSA (2018) considers all common species of probiotics as being safe. Any probiotic microorganism intended for use require an in-depth assessment of their safety. A recent paper (Brodmann et al., 2017) discusses safety evaluation approval for novel food ingredient according to European Union (EU) regulations. Under Regulation 2015/2283 (European Commission, 2015) which came into force on January 1, 2018, the EFSA will perform the scientific risk assessment aimed at facilitating the authorization of novel food. In the U.S. human studies involving probiotics must be conducted within the FDA's framework (Food Drug Administration, 2016). The European qualified presumption of safety (QPS) and the American Generally Recognized as Safe (GRAS) concepts establish a generic risk assessment approach for biological agents. Both concepts are related but differences exist. QPS provides an assessment tool for the EFSA based on reasonable evidence and is responsible for providing the burden of proof while GRAS lays the responsibility on the food business operator. The FDA analyzes every case. Safety studies are performed before efficacy studies can take place, even for widely used probiotics that have a GRAS status, and numerous probiotics are considered as being safe (Guidance for Industry, 2017).

Despite the known level of safety in probiotics there is a significant number of reported cases of *Lactobacillus* strains presumably involved in human infections in consumers of probiotics prior to symptom onset. The published cases, which could display pathogenic potential, mainly affect the elderly, people with immunodeficiency or those who have undergone antitumor therapies, diabetics, and patients with extensive ulcerations of the mucosa of the digestive tract, especially if they have previously been treated with broad-spectrum antibiotics. We have to bear in mind that currently there are no universal accepted guidelines with regard to the administration of probiotics in these patients.

Several metabolic activities present on probiotic lactobacilli which may have an effect on human safety are not treated here, such as the potential ability to synthesize biogenic amines, the production of D-lactate, the deconjugation of bile salts, the presence of β-glucuronidase and glycosidase activities, besides the degradation of hyaluronic acid or platelet aggregation activity, among others.

Most probiotics have been routinely used in products for decades. Therefore, probiotic lactobacilli present no drawbacks for healthy people and no warning of side effects. In conclusion, in the authors' opinion some case reports may be found with a very low frequency in susceptible patients. These types of patients should be properly advised by the doctor or perhaps through health warnings issued on product labeling.

The pathogenic potential of probiotic lactobacilli is quite low, based on their prevalence as normal colonizers of the human body and the low level of infection attributed to them. However, we strongly recommend initiating clinical tests for each of the specific probiotic strains, which may also foster the building of an in-depth body of knowledge on the organism by using molecular testing which should be accomplished under strict conditions in reference laboratories.

Finally, it is important to state that a standard health treatment should not be replaced with probiotics and they should not be taken without advice from a consultant. A probiotic is not an alternative treatment for any health condition. So, please consult your doctor.

## Author Contributions

All authors listed have made a substantial, direct and intellectual contribution to the work, and approved it for publication.

### Conflict of Interest Statement

The authors declare that the research was conducted in the absence of any commercial or financial relationships that could be construed as a potential conflict of interest.
